# Neural Oscillations Reveal Differences in the Process of Word Learning among School-Aged Children from Lower Socioeconomic Status Backgrounds

**DOI:** 10.1162/nol_a_00040

**Published:** 2021-07-13

**Authors:** Julie M. Schneider, Alyson D. Abel, Jacob Momsen, Tina C. Melamed, Mandy J. Maguire

**Affiliations:** University of Delaware, Newark, DE, USA; San Diego State University, San Diego, CA, USA; University of California San Diego, La Jolla, CA, USA; University of Texas at Dallas, Richardson, TX, USA

**Keywords:** theta, beta, alpha, word learning, socioeconomic status, school-age

## Abstract

Building a robust vocabulary in grade school is essential for academic success. Children from lower socioeconomic status (SES) households on average perform below their higher SES peers on word learning tasks, negatively impacting their vocabulary; however, significant variability exists within this group. Many children from low SES homes perform as well as, or better than, their higher SES peers on measures of word learning. The current study addresses what processes underlie this variability, by comparing the neural oscillations of 44 better versus worse word learners (ages 8–15 years) from lower SES households as they infer the meaning of unknown words. Better word learners demonstrated increases in theta and beta power as a word was learned, whereas worse word learners exhibited decreases in alpha power. These group differences in neural oscillatory engagement during word learning indicate there may be different strategies employed based on differences in children’s skills. Notably, children with greater vocabulary knowledge are more likely to exhibit larger beta increases, a strategy that is associated with better word learning. This sheds new light on the mechanisms that support word learning in children from low SES households.

## INTRODUCTION

A child’s ability to learn new words is foundational for subsequent language growth and academic success (Burchinal et al., 2020; Pace et al., 2019). Recent evidence indicates children from lower socioeconomic-status (SES) homes perform well below their peers on measures of word learning, including vocabulary acquisition (Bion et al., 2013; Levine et al., 2020; Maguire et al., 2018; Schwab & Lew-Williams, 2016; Spencer & Schuele, 2012; Weisleder & Fernald, 2013). These group differences in word learning ability mediate SES-related gaps in vocabulary knowledge (Shavlik et al., 2020). Understanding *how* the process of learning a new word differs among children from lower SES households can provide important insights into mechanisms which may serve as compensatory strategies that scaffold later vocabulary growth. The current study addresses this question by investigating changes in the neural oscillations as a word is learned from linguistic context in school-aged children from lower SES households.

Between third and ninth grade children learn around 3,000 new words per year (Wagovich et al., 2012). During this period of rapid vocabulary growth, children are experiencing a shift in vocabulary instruction (Gersten et al., 2010; Nagy & Townsend, 2012). Prior to this point, children often learn new words via more contextualized experiences, such as fast mapping and quick incidental learning (Rice, 1990; Rice et al., 1992), in which one learns the labels for referents in the environment. However, as children progress through school, word learning relies more heavily on decontextualized language, referred to as word learning from linguistic context, in which the child must use only the surrounding language to guide their learning (Elleman et al., 2019). This type of learning is an incremental process that includes identifying a new word, holding potential word meanings in memory, and eliminating incorrect meanings as new information becomes available (Fukkink et al., 2001; Nagy et al., 1985). Difficulty with one or more aspects of this process could negatively impact a child’s ability to learn the meaning of the new word.

Recent work has revealed that children from lower SES households face difficulties with this type of word learning, even when they know all the surrounding words in the sentence (Maguire et al., 2018). These findings suggest that the reason children from lower SES households have difficulty learning new words is more likely related to the process of learning, rather than knowledge about individual word meanings. To better understand what is different about the process of word learning from context between children from lower and higher SES homes, Ralph et al. (2020) examined changes in the N400 event related potential (ERP) component as a word was learned. The authors found that, similar to past reports (Abel et al., 2018; Mestres-Missé et al., 2007), children from higher SES homes exhibited a significant attenuation of the N400 as a word was learned. Interestingly, children from lower SES households showed no N400 attenuation, despite still learning the novel word. Significant variability existed within the lower SES sample, indicating some children may be significantly better word learners than others. While the authors speculated that the lack of N400 attenuation may be associated with the depth and breadth of semantic networks, or different, diffuse patterns of neural engagement, it remains unclear what compensatory neural process children from lower SES households engage when successfully learning a word from linguistic context.

New insights about the process of word learning may be revealed by analyzing event related spectral perturbations (ERSPs) within the EEG signal. Differences observed in frequency power changes, which result from ERSP analyses, may provide additional information about the underlying neural mechanisms being engaged (Davidson & Indefrey, 2007; Schneider & Maguire, 2018b). Changes in the theta, alpha, beta, and gamma frequency bands have been implicated across numerous studies of language processing in adults, resulting in a growing consensus that different linguistic tasks result in activation (or deactivation) within specific frequency bands. Although a single frequency band may be associated with multiple cognitive processes, a thorough review by Prystauka and Lewis (2019) synthesized current ERSP research, revealing the following well-supported relationships during language processing tasks. Increases in theta power are often associated with lexical retrieval and increased working memory load during integration of new information with the preceding sentence and discourse context (Bastiaansen & Hagoort, 2015; Bastiaansen et al., 2002; Hagoort et al., 2004; Schneider et al., 2018; Schneider et al., 2016; Wang, Zhu, & Bastiaansen, 2012). Decreases in both alpha and beta power simultaneously correspond to processing of new linguistic information and the demands this new information has upon working memory (Sauseng et al., 2005; Schneider et al., 2018; Wang, Jensen et al., 2012). Alternatively, increases in beta reflect syntactic unification operations (Bastiaansen et al., 2010; Kielar et al., 2014; Kielar et al., 2015; Lewis et al., 2015; Schneider & Maguire, 2018a; Schneider et al., 2016), while increases in gamma reflect semantic unification (Bastiaansen & Hagoort, 2015; Fedorenko et al., 2016; Lewis & Bastiaansen, 2015). Based on the substantial evidence implicating the role of theta, alpha, beta, and gamma oscillations in language processing, the current study investigates changes in these frequencies as a word is learned within a low SES population.

In the current study we examine neural oscillations to clarify which neural processes children from lower SES households engage when successfully learning a word from linguistic context. To better understand which processes underlie variability in word learning within a low SES population, we will compare the neural activation engaged by children who performed well on the task with that of individuals from similar households who performed poorly on the task. To better elucidate why vocabulary is so strongly associated with word learning (Maguire et al., 2018; Shavlik et al., 2020), we will determine if differences in the ERSP markers measured during the word learning task are associated with vocabulary knowledge. These findings can inform us about the types of strategies lower SES children engage to successfully learn a word, whether those strategies differ depending on word learning abilities, and if such differences are associated with greater vocabulary knowledge.

## METHODS

### Participants

We investigated neural changes in the ERSPs of 44 children ages 8–15 years old raised in lower SES households. We use maternal education as a proxy for SES in the current study, where all mothers self-reported having a high school diploma/GED or lower. These children came from a larger dataset (*N* = 275) of 8–15-year-olds from a variety of SES backgrounds. In this larger sample, which included children from low and high SES households, the median word learning performance was 66% correct (*SD* = 20.26%, Range [12%–98%]). Learner status was determined via a median split: Worse learners performed below the median, while better learners performed at or above the median. The low SES subset investigated in the current analysis included 22 children who, as a group, performed significantly above the group-median on the word learning task (Better Learners; *t*(21) = 6.28, *p* < 0.001), and 22 children who, as a group, performed significantly below the group-median on the word learning task (Worse Learners; *t*(21) = −8.39, *p* < 0.001). Groups did not significantly differ in dual language experience, age, vocabulary knowledge, working memory or reading ability. Full demographic information for each group can be found in Table 1.

**Table 1.** Demographic information for children classified as better or worse learners

	**Better learners**	**Worse learners**	***p* value**
*N*	22	22	
Gender (F:M)	11:11	17:5	0.117
Age [mean (range)]	12.68 [8–15]	11.45 [8–15]	0.073
Dual language experience (# with bilingual exposure)	17	19	0.696

Maternal education			
Less than HS Diploma	10	10	
HS GED/Diploma	12	12	

Average annual household income [mean (range)]	$42,455 ($10,000–135,000)	$33,273 ($17,013–82,500)	0.25
Income-to-needs ratio [mean (*SD*)]	2.02 (1.62)	1.39 (0.73)	0.11
Reading ability GORT-ORI [mean (*SD*)]	93.77 (13.23)	88.43 (11.77)	0.17
Vocabulary knowledge PPVT-4; [mean (*SD*)]	98.9 (12.13)	92.55 (12.22)	0.09
Working memory digit span; [mean (*SD*)]	8.23 (2.35)	7.59 (1.59)	0.3
Word learning accuracy			
[mean (*SD*)]	75% (7%)	51% (9%)	<0.001
[range]	[66–92%]	[38–64%]	

### Stimuli and Procedure

Parental consent and child assent were obtained prior to participation in the task in accordance with the Institutional Review Board at the University of Texas at Dallas, the Good Clinical Practice Guidelines, the Declaration of Helsinki, and the U.S. Code of Federal Regulations.

After assent and consent were obtained, parents or guardians provided information about their child including medical history, language history related to dual language experience, handedness, and neurological history. Participants completed the Edinburgh Handedness Inventory (Oldfield, 1971) to verify right-handedness before completing the behavioral and EEG testing session. Children then completed a battery of behavioral assessments and the word learning from context task, as described in greater detail below. Parents and children received a $50 gift card for their participation in the study.

#### Socioeconomic status

SES is multifaceted, requiring multiple data points, associated with income status, maternal education, and other household structure measures (Entwislea & Astone, 1994). While research can use multiple separate indicators, a composite index, or just one single scale to quantify SES, the most often-used, single-scale indicator in recent child development research has been maternal education (Bornstein et al., 2003; Bradley & Corwyn, 2002; Campbell et al., 2003; Coleman, 2009; Davis-Kean, 2005; DeGarmo et al., 1999; Dollaghan et al., 1999; Ensminger & Fothergill, 2014; Harding et al., 2015; Hoffman, 2003; Richels et al., 2013). Reasons for this may be due to ease of data collection and reliability of information, participants’ reluctance to provide income information, and the instability of some components of SES—such as parental occupation and income, which can fluctuate—while parental education levels tend to be stable (Duncan & Magnuson, 2003). Across all of the studies listed above, maternal education consistently emerges as the most robust predictor of child outcomes. Therefore, the current study utilizes maternal education as a proxy for SES. Specifically, we investigated children who came from families where mothers had obtained a high school diploma or GED, or less, but had not pursued any post-secondary education. We believe this accurately captures a lower SES background, as research in the United States has shown that there is more than a 0.5-standard deviation difference in test scores between children whose parents have a college degree and children whose parents have a high school degree (Dollaghan et al., 1999; Duncan & Magnuson, 2012). In fact, Hoff (2003) compared households where parents had completed high school to parents who had completed college and identified SES-related differences in maternal speech and in child language development outcomes. Children of mothers who had completed high school, but had not completed college, were associated with poorer language outcomes, than children of mothers who had completed college.

#### Language history

All children were required to attend schools where instruction was provided in English only. Although groups in the current study did not significantly differ in their composition of bilingual and monolingual individuals (see Table 1), groups did consist primarily of children with bilingual Spanish-English language experiences. This is because the location in which the study was conducted, Dallas, Texas, is primarily Hispanic (Texas Demographic Center, n.d.). And while a high percentage of Dallas County’s population lives two times below the federal poverty line (U.S. Census Bureau, n.d.), Hispanic and Black families are disproportionately more likely to live in poverty than White and Asian families. Specifically, the median household income for Hispanic families in Dallas is around $40,500, as compared to $68,800 for White families (U.S. Census Bureau, n.d.). Therefore, to measure the bilingual language experiences of children in the current study, we asked parents to report which languages their child spoke and the age at which their child began speaking each language. All bilingual speakers were English-Spanish bilinguals, and to determine which language children spoke first, we subtracted the age Spanish was learned from the age English was learned. Therefore, more negative numbers indicated English was learned first, positive numbers indicated Spanish was learned first, and zero would indicate the child was a simultaneous bilingual.

We next asked parents how well (on a scale of 1–5) their child could use each of the following six domains within each language: speaking, listening, writing, reading, grammar, pronunciation. An average score was computed across all six domains per language to obtain a general understanding of the child’s language experience. A five would indicate expertise, whereas a one would indicate little to no experience. To compute which language children were more experienced in, we subtracted their average Spanish experiences from their average English experiences. Counter to the above comparison, a more positive number would indicate higher English competency, while a more negative score would indicate higher Spanish competency. A score close to zero would indicate children were highly competent in both languages. As can be seen in Table 2, better and worse learners did not significantly differ in the age at which English was learned, nor did they significantly differ in their general understanding of the English language based on parental self-report. Groups also did not significantly differ in their reading ability or vocabulary knowledge on normed assessments given in English (Table 1), suggesting they were relatively evenly matched in their knowledge of the English language. However, to test whether an effect of bilingual language experience influenced vocabulary, we included this in the multiple regression analysis.

**Table 2.** Bilingual language experiences for children classified as better or worse learners

	**Better learners**	**Worse learners**	***p* value**
Dual language experience (# with bilingual exposure)	17	19	0.696
Average age English was learned compared to Spanish [mean (*SD*)]	0.62 (3.77)	2.68 (2.99)	0.08
Average English competency compared to Spanish [mean (*SD*)]	0.38 (1.86)	0.02 (1.44)	0.52

*Note*. More positive numbers for age language learned indicates children were older when they learned English, as compared to Spanish. More positive numbers for competency indicate children were more experienced with English than Spanish, although scores near zero indicate high competency in both languages.

#### Behavioral assessments

Behavioral assessments included the Peabody Picture Vocabulary Test (PPVT-4; Dunn & Dunn, 2007) to assess static receptive vocabulary knowledge, the Gray Oral Reading Test (GORT-5; Wiederholt & Bryant, 2012) to assess reading fluency and comprehension, and a reverse digit span task as a measure of working memory ability.

#### Word learning from context task

The current experimental task has been used in previous studies with children (Abel et al., 2018; Maguire et al., 2018; Ralph et al., 2020). Children read 100 sets of sentence triplets presented one word at a time on a computer screen. Stimuli included only early-acquired, high frequency words expected to be within the child’s vocabulary per the MacArthur–Bates Communicative Development Inventory (CDI; Fenson et al., 1994). Sentences contained between six and nine words and the last word of each sentence was a novel pseudoword, referred to as the target word. Target words were placeholders for concrete, early-acquired nouns (Fenson et al., 1994). These words were phonologically possible, monosyllabic consonant-vowel-consonant words (Storkel, 2013). Table 3 shows an example of a sentence triplet.

**Table 3.** Example sentence triplet

**Sentence 1** (low cloze probability)	Her parents bought her a *pav*.
**Sentence 2** (medium cloze probability)	The sick child spent the day in his *pav*.
**Sentence 3** (high cloze probability)	Mom piled the pillows on the *pav*.

*Note*. The nonword “pav” stood for the noun “bed.”

There were two conditions: one in which children could attach meaning to the target (50 trials) and a control condition (50 trials). In the condition in which children could attach meaning, all three sentences scaffolded meaning acquisition for the target word, and cloze probability of the target word increased across the presentation of the three sentences. The control condition did not increase cloze probability across the sentence triplet, and there was no semantically plausible word that could take the place of the target word in the sentence triplet. After each sentence triplet, the examiner prompted participants to verbally report whether there was a word that could replace the target word, and what that word could be (if they responded affirmatively). Since this study sought to examine differences in successful word learning from context, we focused only on sentence triplets in which children successfully mapped meaning to the target word. Incorrect responses and trials related to the control condition were not included in subsequent analyses (information related to processing of these other conditions can be found in Abel et al., 2018).

Upon completion of the behavioral assessments, EEG testing began. Participants sat in a comfortable chair in a sound attenuated booth about three feet from a computer monitor. Prior to the task, participants completed a training set for which they were given accuracy feedback. Once the task began, participants did not receive any feedback. Children were randomly assigned to one of eight presentation conditions in which sentence triplet order was randomized.

### EEG Pre-Processing

EEG was recorded with a 62-channel EEG system (CURRY, Compumedics Neuroscan) which features an online sampling rate of 1000 Hz. Electrode impedances were kept below 10 kΩ. Recordings were online referenced to an electrode between Cz and CPz. All data was saved using CURRY Neuroimaging Suite software and analyzed within MATLAB.

After recording, continuous data was high-pass filtered at 0.1 Hz, low-pass filtered at 50 Hz, and re-referenced to the average across the entire scalp. An independent components analysis (Delorme et al., 2001) was carried out. Components related to eye-movements or muscle activity were identified and removed from the data on the basis of their time courses, frequency spectra, and topographies using the multiple artifact rejection algorithm plug-in (Winkler et al., 2014; Winkler et al., 2011). The data was then epoched from 500 ms before to 1,000 ms after target word onset. Remaining artifactual epochs were removed after visual inspection and trials in which the participant gave an incorrect response were removed as well. Only trials related to correct responses were kept as the current study is interested in the neural mechanisms engaged during successful learning; however, because we are comparing individuals who did better and worse on the task, groups differed in the number of trials retained. On average, better word learners had 38.32 (*SD* = 5.13) and worse learners had 25.64 (*SD* = 7.33) remaining trials during presentation of the target word in sentence 1 (*t*(42) = 6.65, *p* < 0.001). During presentation of the target word in sentence 3, better word learners had on average 38.36 (*SD* = 5.15) remaining trials and worse word learners had 25.64 (*SD* = 7.33) remaining trials (*t*(42) = 6.75, *p* < 0.001). While large differences in trial count (~30 trials or more) were not present, differences in trial count between groups can introduce a positive bias for the group with more trials, as raw power values can only be positive and thus noise is more likely to increase than decrease power (Cohen, 2014).

We took two approaches to check whether sufficient trials were retained in the ERSP data: (1) If data are clean, no outliers exist, and a sufficient number of trials are retained, the mean and median of dB power should produce similar results. (2) The reliability of trial-averaged power can be estimated using random subsets of trials, and if no noise is present in the data, the time course of frequency-band-specific power on one trial will correlate perfectly with the time course of frequency-band-specific power averaged over all trials. This analysis can be repeated over frequencies, so that the resulting map of correlation coefficients is a frequency-by-trial count map (Cohen, 2014). Approach 1 yielded strong correlations between the mean and median of dB power for both worse (*R* = 0.51) and better (*R* = 0.74) learners (Supplementary Figure 1). Approach 2 was conducted at electrode CP1 and results indicate that approximately 25 trials, corresponding to a correlation of around 0.7, was sufficient across all frequencies per group (Supplementary Figure 2; supporting information can be found online at https://www.mitpressjournals.org/doi/suppl/10.1162/nol_a_00040). After confirming that the number of trials retained was sufficient, epoched EEG data was baseline corrected by subtracting neural activation from the 500 ms time window prior to the onset of the target word.

### ERSP Analysis

Whereas ERPs reflect phase-aligned information only, ERSP analysis results in a time-resolved measure of spectral power that is then averaged over trials, reflecting oscillatory activity regardless of whether it is phase-aligned. In the current study, time-frequency transformation of the EEG data was performed using handwritten MATLAB scripts that build upon Fieldtrip functions (Oostenveld et al., 2011; all scripts are publicly available at https://github.com/juliagoolia28/manuscripts/tree/master/eeg_learner). Data was convolved using a Hanning taper from 3 to 80 Hz in steps of 0.5 Hz.

To compare the change in neural activity due to learning across the two groups, we tested the difference between ERSP measures at the onset of the first and third sentence target word between better and worse word learners. Statistical comparisons were made using a non-parametric cluster-based permutation at an alpha cluster threshold of 0.05 (Maris & Oostenveld, 2007). This permutation controls for the multiple comparisons problem created by the large number of location, time, and frequency points in the ERSP data. This approach is particularly useful for analyses in which there are few prior hypotheses regarding the nature and time course of the effects of interest. To investigate the interaction between group and sentence, we first computed a difference matrix within each group by doing a point-by-point subtraction of the data between the third and first target word onset, and then computed a cluster-based permutation on the difference between those two matrices, within each frequency band of interest (theta, 4–8 Hz; alpha, 9–12 Hz; beta, 13–30 Hz; and gamma, 30–80 Hz). Cluster-level statistics were derived from the summed adjacent *t* values computed from the randomized permutation procedure described above (*N* = 1,000). Cluster neighbors were defined using a triangulation method. As such our analyses estimate the time across the entire 1 s window (target word onset − 1,000 ms after), where spectral dynamics were different between groups.

## RESULTS

### ERSP Results

**Figure 1.**
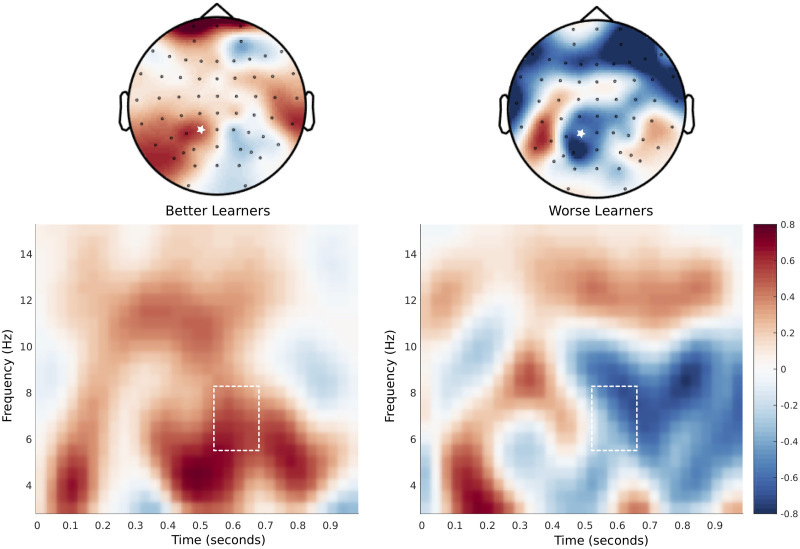
Change in theta activation from sentences 1 to 3 in better and worse word learners. Red represents an increase in activation, while blue denotes decreases in activation. Scalp maps are averaged over the time window of group differences estimated by the cluster-based analysis (0.556 to 0.652 s), while spectrograms represent change in activation over time at electrode P1 (starred on the scalp map). The white dashed rectangles highlight significant activation identified by the cluster analysis.

The nonparametric cluster-based permutation analysis indicated an effect of condition (*p* < 0.05) within the theta band (6–8 Hz; *F*_cluster_ = 78.66, *p* < 0.001). This corresponded to a cluster at central-parietal electrodes (CZ, C2, CP1, CPZ, CP2, P1; Figure 1) from 0.556 to 0.652 s after target word onset. Better learners demonstrated an increase in theta power from sentence 1 to 3 (*M* theta change = 0.29, *SD* = 0.52).

**Figure 2.**
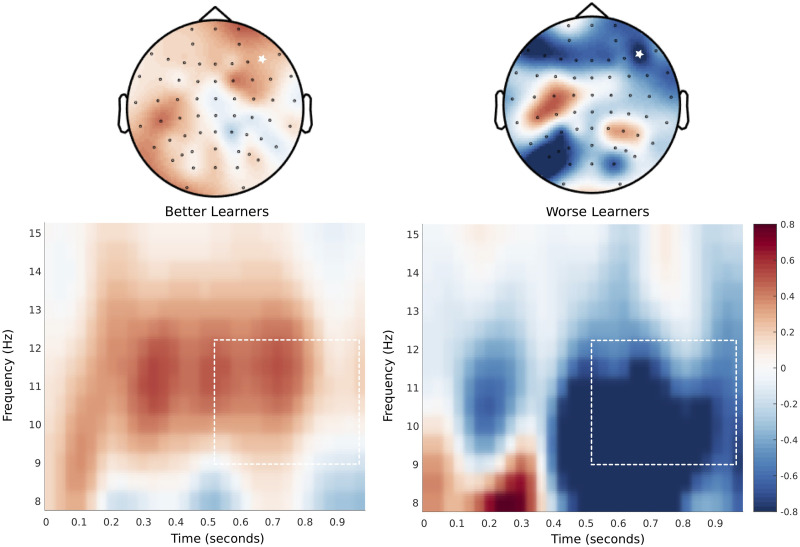
Change in alpha activation from sentences 1 to 3 in better and worse word learners. Red represents an increase in power, while blue denotes decreases in activation. Scalp maps are averaged over the time window of estimated group differences (0.524 to 0.972 s), while spectrograms represent change in activation over time at electrode F6 (starred on the scalp map). The white dashed rectangles highlight significant activation identified by the cluster analysis.

A cluster in the observed data was also found in the alpha band (9–12 Hz; *F*_cluster_ = 1186.81, *p* < 0.001; Figure 2) across widespread electrodes (AF4, F4, F6, F8, FC6, FT8, CZ, C2, T8, CP1, CPZ, CP2, CP4, TP8, PZ, P2, P4, P6, P8, POZ, PO4, PO6, PO8, O2, CB2) from 0.524 to 0.972 s after target word onset. Worse learners demonstrated greater alpha decreases from sentence 1 to 3 (*M* alpha change = −0.51, *SD* = 0.91).

**Figure 3.**
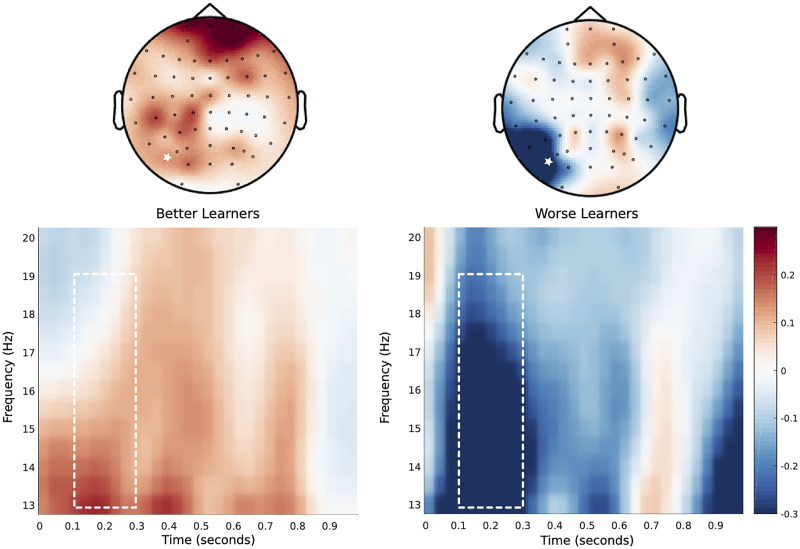
Change in beta activation from sentences 1 to 3 in better and worse word learners. Red represents an increase in activation, while blue denotes decreases in activation. Scalp maps are averaged over the time window of estimated group differences (0.108 to 0.3 s), while spectrograms represent change in activation over time at electrode P7 (starred on the scalp map). The white dashed rectangles highlight significant activation identified by the cluster analysis.

There was also a cluster in the observed data in the lower beta band (13–19 Hz; *F*_cluster_ = 694.79, *p* < 0.001; Figure 3) at left parietal and occipital electrodes (TP7, CP5, P7, P5, P3, P1, PO7, PO5, PO3, O1) from 0.108 to 0.3 s after target word onset. Better learners demonstrated an increase in beta power from sentence 1 to 3 (*M* beta change = 0.02, *SD* = 0.19).

While a cluster was observed in the data in the gamma band (48–51 Hz; *F*_cluster_ = 84.51, *p* = 0.87) at frontal electrodes (FP1, FPz, AF3, AF4) between 0.94 and 0.97 s after target word onset, it did not reach the threshold for significance.

### Regression Results

Since previous studies indicate that vocabulary knowledge mediates SES-related gaps in word learning (Maguire et al., 2018), and that word learning ability mediates SES-related gaps in vocabulary (Shavlik et al., 2020), we sought to clarify which mechanisms underlying word learning are associated with greater vocabulary knowledge. Although groups did not significantly differ in static vocabulary knowledge, as measured by the PPVT-4, we utilized multiple regression analyses to determine whether differences in the neural mechanisms engaged during word learning were associated with individual variability in vocabulary knowledge, regardless of the directionality of that relationship. For each individual (*N* = 44), we extracted their mean amplitude of activation within each significant cluster (change in EEG response to target word between the first and third sentence). We then computed a multiple regression with vocabulary knowledge (PPVT-4) as the outcome variable. Age, gender, and dual language experience were included as covariates. Individual mean amplitude of theta, alpha, and beta, in their respective clusters, were added as predictor variables. Reading ability (GORT-ORI) and working memory (Digit Span) were also included in the model as covariates.

In this model, age (*B* = −1.98, *p* = 0.02) and reading ability (*B* = 0.59, *p* < 0.001) accounted for significant variance in vocabulary knowledge. Importantly though, increases in beta activation between when a word is first encountered and when the child has attached meaning to that word accounted for a significant proportion of variability in vocabulary knowledge (*B* = 13.82, *p* = 0.01). A greater change in beta activation from sentence 1 to 3 during word learning is associated with stronger vocabulary knowledge, regardless of age, gender, dual language experience, working memory, or reading ability. This was not the case for changes in theta (*B* = −4.54, *p* = 0.18) or alpha power (*B* = 3.18, *p* = 0.28) from sentence 1 to 3 (see Table 4).

**Table 4.** Neural correlates during word learning regressed on vocabulary

	**β**	***SE***	***t* value**	***p* value**
(Intercept)	65.23	15.85	4.12	0.00**
Age	−1.98	0.81	−2.43	0.02*
Gender	0.70	3.13	0.22	0.83
Dual language experience	−0.74	3.76	−0.20	0.85
Working memory	0.37	0.80	0.46	0.65
Reading ability	0.59	0.13	4.70	0.00**
Theta	−4.54	3.32	−1.37	0.18
Alpha	3.18	2.86	1.11	0.28
Beta	13.82	5.27	2.62	0.01*

*Note*. *p < 0.05, **p < 0.001.

## DISCUSSION

Establishing a better understanding of the nature of differences in early vocabulary development, especially in a low SES population that is known to struggle disproportionately with learning new words, is critical for creating subsequent interventions that promote word learning success in children from all SES backgrounds (Shavlik et al., 2020). In the current study, we found that children who are stronger word learners engage theta and beta when learning a new word, while children who are not as strong word learners engage more alpha. Greater increases in beta were further associated with greater vocabulary knowledge, suggesting this neural mechanism underlies a strategy that supports both successful word learning and vocabulary. Critically, consistent with recent research, our findings suggest that poorer word learning and vocabulary outcomes among children from lower SES households are not attributed to differences in vocabulary alone, as groups did not significantly differ in vocabulary, but are rather associated with differences in the skills and strategies used to build that vocabulary (Levine et al., 2020; Shavlik et al., 2020). Some children from lower SES households are better word learners, while others are not, and these differences are related to the process of *how* a word is learned.

Across the course of learning a word in the current task, children who were better word learners engaged beta more than children who were worse word learners. These increases in beta have been thought to reflect syntactic unification operations (Bastiaansen et al., 2010; Kielar et al., 2014; Kielar et al., 2015; Lewis et al., 2015; Schneider & Maguire, 2018a; Schneider et al., 2016), suggesting that school-aged children who integrate syntactic information with greater ease are better word learners. As previous reports have shown, enhanced beta activation during comprehension of naturally paced sentences improves with age and is related to improved integration of sentence level information (Schneider et al., 2018). Therefore, it is possible that the lack of beta activation in worse word learners represents a maturational lag. This critical difference in beta engagement is then associated with variability in measures of static vocabulary, as indicated by our regression analyses.

Two possible explanations may account for the relationship between vocabulary and increases in beta during word learning. First, it possible that children who have greater existing vocabulary knowledge are better equipped with the skills necessary to engage beta more effectively. In support of this explanation, Maguire et al. (2018) found that the relationship between SES and word learning was mediated by vocabulary. The other possible reason for this association is that increases in beta underlie effective word learning which leads to larger vocabulary knowledge over time. This interpretation is supported by work by Shavlik et al. (2020), which found SES-related gaps in vocabulary were mediated by word learning ability. Both interpretations promote theories which suggest “skill begets skill” (Heckman, 2006), and point to different, but not mutually exclusive, pathways, by which the vocabulary gap may grow between children from lower and higher SES households during the school years.

This study also uncovered differences in theta and alpha engagement between better and worse word learners from low SES households. For children who performed better on the word learning task, a theta increase occurred from sentence 1 to sentence 3, while children who performed more poorly exhibited greater alpha power decreases from sentences 1 to 3. Given that previous literature on sentence processing has related increases in theta to lexical retrieval and increased working memory load during integration of new information with the preceding sentence and discourse context (Bastiaansen et al., 2002; Bastiaansen & Hagoort, 2015; Hagoort et al., 2004; Schneider et al., 2018; Schneider et al., 2016; Wang, Zhu, & Bastiaansen, 2012), our findings suggest that engagement of this cognitive process is beneficial for word learning. Alternatively, decreases in alpha, which are often associated with increased working memory demands to process new linguistic information (Sauseng et al., 2005; Schneider et al., 2018; Wang, Jensen et al., 2012), aid word learning, but are not the best strategy when learning a new word. Alpha power decreases may therefore represent a compensatory strategy engaged to learn a new word when difficulty integrating lexical and syntactic information in the sentence context exists. Bolstering the cognitive skills supported by a greater theta and beta increase could lead to better word learning outcomes for children who currently have difficulty learning new words from linguistic context.

Given the multifaceted nature of SES, the current study sought to disentangle why some children, coming from similar households, raised in similar environments, learn words better than other children. For this reason, we have only focused on differences in word learning among children from lower SES households. While it remains unclear whether good word learners from lower SES households engage the same neural processes as those from higher SES households, it is clear that the process good word learners from lower SES households engage to learn a word is successful. This is because, behaviorally, good word learners from lower SES households learned just as many, if not more, words on average than their higher SES peers. Differences in spontaneous brain oscillations in baseline EEG on the basis of SES is also a reason we elected to compare children from similar environments. In typical brain development, spontaneous brain oscillations result in decreases in low frequency rhythms, such as theta, and increases in higher frequency rhythms, such as alpha and gamma (Anderson & Perone, 2018; Harmony et al., 1988; Uhlhaas et al., 2010). However, research has shown that children from lower SES households exhibit more theta power, and reduced alpha/gamma power (Brito et al., 2016; Brito et al., 2020; Maguire & Schneider, 2019; Tomalski et al., 2013). These EEG differences in children from lower SES homes are generally interpreted as a developmental lag due to an inability to meet basic physiological needs and are associated with differences in cognitive and language outcomes (Brito et al., 2020; Maguire & Schneider, 2019; Otero et al., 2003; Vanderwert et al., 2016). By comparing children from similar environments (see Table 1 and Supplementary Table 1), the current study sought to limit the influence that differences in spontaneous brain oscillations in baseline EEG may have upon our results. Taking this focused approach limits our ability to speculate as to whether the strategies engaged during word learning here are exclusive to children from low SES homes or might also extend to children from higher SES homes.

It is important to note that the current study does not identify the origins of differences in word learning ability. Here, we focus upon differences between children from low SES homes, as measured by maternal education; however, it would be beneficial for future research to extend this work and determine whether more proximal measures of the child’s environment, such as the home language environment, stress, and/or nutrition, account for variability in word learning. It is also possible that individual differences in the learner, associated with IQ or executive function, may account for variability in word learning, which the current study did not measure. In future studies, we intend to include more extensive measures of vocabulary, reading, IQ, and executive function to more clearly tease apart which mechanisms may account for additional variability in word learning skill between groups.

Our sample was highly representative of ethnicity in Dallas, Texas (where the study was conducted), and therefore consisted of primarily bilingual English-Spanish speakers. While group differences in neural engagement persisted, independent of the influence of bilingual exposure, it cannot be overlooked that monolingual speakers may engage a different cognitive process when learning a new word. It is important to consider the quantity of children’s exposure to each language: Bilingual children who hear a large amount of a particular language learn more words and grammar in that language (Hoff et al., 2012) and show more efficient processing of that language (Byers-Heinlein & Lew-Williams, 2013; Fernald et al., 2013). Based on parental report the two groups did not differ in exposure to both languages, although on average, worse learners were more likely to learn English later than better learners. Thus, better learners may have included more simultaneous bilinguals and worse learners could have included more successive bilinguals. Nonetheless, all children in the current study attended schools where instructions and learning occur in English, and recent reports have indicated that dual language learners are equally likely to learn words in both English and Spanish (Pace et al., 2021; Luo et al., 2021). Thus, we believe our findings still have important implications about the attributes that are most important for these children when learning in the classroom. Ages 8–15 years also represent a relatively broad age range, when changes occur both within children and within the school curriculum that can affect word learning. While we aimed to address much of this age-related variation by matching across groups and controlling for age in all statistical analyses, future research should investigate developmental differences in word learning ability within a low SES sample. Lastly, the relationship between neural oscillations and cognitive processes in the current study is based on current interpretations in the field; however, more work needs to be done to substantiate these relationships in children.

One of the most central takeaways from this study is in the acknowledgement that there is significant variability in word learning performance among children from low SES backgrounds. Namely, that not all children raised in low SES environments are bound to have poor word learning or vocabulary outcomes. Median word learning performance was determined in a larger sample, including children from higher SES backgrounds. Therefore, children who were identified as better word learners in the current low SES sample, were accurately identifying words similar to their higher SES peers. While we did not directly compare word learning ability in children from both low and high SES households, our findings suggest increases in beta as a word is learned is an effective strategy for promoting a child’s vocabulary knowledge. These findings may not be restricted to low SES children only, but rather, may generalize to children across SES strata. Therefore, it would be fruitful for interventions to target the process of word learning, and strategies related to the syntactic unification of information during word learning, when seeking to ameliorate gaps in vocabulary knowledge.

## FUNDING INFORMATION

Julie M. Schneider, Directorate for Social, Behavioral and Economic Sciences (https://dx.doi.org/10.13039/100000088), Award ID: 1911462. Mandy J. Maguire, Directorate for Social, Behavioral and Economic Sciences (https://dx.doi.org/10.13039/100000088), Award ID: 1551770.

## AUTHOR CONTRIBUTIONS

**Julie M. Schneider**: Conceptualization; Methodology; Formal analysis; Writing – original draft; Visualization; Project administration; Funding acquisition. **Alyson D. Abel**: Methodology; Writing – review & editing; Funding acquisition. **Jacob Momsen**: Data curation; Validation; Writing – review & editing. **Tina C. Melamed**: Writing – review & editing. **Mandy J. Maguire**: Methodology; Writing – review & editing; Funding acquisition; Supervision; Resources.

## TECHNICAL TERMS

Neural oscillations: Brain waves produced by synchronized groups of neurons communicating with each other across the scalp.
N400 event related potential (ERP): A negative-going deflection in the averaged EEG signal that occurs around 400 ms after a stimulus and is related to semantic processing.
Event related spectral perturbations (ERSPs): Changes in brainwaves in response to a stimulus across frequency bands that delineate slow, moderate, and fast waves.
Cloze probability: The probability or likelihood that a target word will complete a particular sentence.

## References

[bib1] Abel, A. D. , Schneider, J. M. , & Maguire, M. J. (2018). N400 response indexes word learning from linguistic context in children. Language Learning and Development, 14(1). 10.1080/15475441.2017.1362347

[bib2] Anderson, A. J. , & Perone, S. (2018). Developmental change in the resting state electroencephalogram: Insights into cognition and the brain. Brain and Cognition, 126, 40–52. 10.1016/j.bandc.2018.08.001, 30144749

[bib4] Bastiaansen, M. , & Hagoort, P. (2015). Frequency-based segregation of syntactic and semantic unification during online sentence level language comprehension. Journal of Cognitive Neuroscience, 27(11), 2095–107. 10.1162/jocn_a_00829, 26042498

[bib5] Bastiaansen, M. , Magyari, L. , & Hagoort, P. (2010). Syntactic unification operations are reflected in oscillatory dynamics during on-line sentence comprehension. Journal of Cognitive Neuroscience, 22(7), 1333–1347. 10.1162/jocn.2009.21283, 19580386

[bib3] Bastiaansen, M. C. M. , van Berkum, J. J. A. , & Hagoort, P. (2002). Event-related theta power increases in the human EEG during online sentence processing. Neuroscience Letters, 323(1), 13–16. 10.1016/S0304-3940(01)02535-6, 11911979

[bib6] Bion, R. A. H. , Borovsky, A. , & Fernald, A. (2013). Fast mapping, slow learning: Disambiguation of novel word-object mappings in relation to vocabulary learning at 18, 24, and 30 months. Cognition, 126(1), 39–53. 10.1016/j.cognition.2012.08.008, 23063233 PMC6590692

[bib7] Bornstein, R. F. , Languirand, M. A. , Geiselman, K. J. , Creighton, J. A. , West, M. A. , Gallagher, H. A. , & Eisenhart, E. A. (2003). Construct validity of the Relationship Profile Test: A self-report measure of dependency-detachment. Journal of Personality Assessment, 80(1), 64–74. 10.1207/S15327752JPA8001_15, 12584069

[bib8] Bradley, R. H. , & Corwyn, R. F. (2002). Socioeconomic status and child development. Annual Review of Psychology, 53, 371. 10.1146/annurev.psych.53.100901.135233, 11752490

[bib9] Brito, N. H. , Fifer, W. P. , Myers, M. M. , Elliott, A. J. , & Noble, K. G. (2016). Associations among family socioeconomic status, EEG power at birth, and cognitive skills during infancy. Developmental Cognitive Neuroscience, 19, 144–151. 10.1016/j.dcn.2016.03.004, 27003830 PMC4912880

[bib10] Brito, N. H. , Troller-Renfree, S. V. , Leon-Santos, A. , Isler, J. R. , Fifer, W. P. , & Noble, K. G. (2020). Associations among the home language environment and neural activity during infancy. Developmental Cognitive Neuroscience, 43, 100780. 10.1016/j.dcn.2020.100780, 32510343 PMC7200831

[bib11] Burchinal, M. , Foster, T. J. , Bezdek, K. G. , Bratsch-Hines, M. , Blair, C. , & Vernon-Feagans, L. (2020). School-entry skills predicting school-age academic and social–emotional trajectories. Early Childhood Research Quarterly, 51, 67–80. 10.1016/j.ecresq.2019.08.004

[bib12] Byers-Heinlein, K. , & Lew-Williams, C. (2013). Bilingualism in the early years: What the science says. LEARNing landscapes, 7(1), 95. 10.36510/learnland.v7i1.632, 30288204 PMC6168212

[bib13] Campbell, T. F. , Dollaghan, C. A. , Rackette, H. E. , Paradise, J. L. , Feldman, H. M. , Shriberg, L. D. , Sabo, D. L. , & Kurs-Lasky, M. (2003). Risk factors for speech delay of unknown origin in 3-year-old children. Child Development, 74(2), 346–357. 10.1111/1467-8624.7402002, 12705559

[bib14] Cohen, M. X. (2014). Analyzing neural time series data: Theory and practice. MIT Press. 10.7551/mitpress/9609.001.0001

[bib15] Coleman, J. S. (2009). Social capital in the creation of human capital. American Journal of Sociology, 94, S95–S120. 10.1086/228943

[bib16] Davidson, D. J. , & Indefrey, P. (2007). An inverse relation between event-related and time-frequency violation responses in sentence processing. Brain Research, 1158, 81–92. 10.1016/j.brainres.2007.04.082, 17560965

[bib17] Davis-Kean, P. E. (2005). The influence of parent education and family income on child achievement: The indirect role of parental expectations and the home environment. Journal of Family Psychology, 19(2), 294–304. 10.1037/0893-3200.19.2.294, 15982107

[bib18] DeGarmo, D. S. , Forgatch, M. S. , & Martinez, Jr., C. R. (1999). Parenting of divorced mothers as a link between social status and boys’ academic outcomes: Unpacking the effects of socioeconomic status. Child Development, 70(5), 1231–1245. 10.1111/1467-8624.00089, 10546342

[bib19] Delorme, A. , Makeig, S. , & Sejnowski, T. (2001). Automatic artifact rejection for EEG data using high-order statistics and independent component analysis. In Proceedings of the 3rd International Independent Component Analysis and Blind Source Decomposition Conference (vol. 457, p. 462), December 9–12, San Diego, CA.

[bib20] Dollaghan, C. A. , Campbell, T. F. , Paradise, J. L. , Feldman, H. M. , Janosky, J. E. , Pitcairn, D. N. , & Kurs-Lasky, M. (1999). Maternal education and measures of early speech and language. Journal of Speech, Language, and Hearing Research, 42(6), 1432–1443. 10.1044/jslhr.4206.1432, 10599625

[bib21] Duncan, G. J. , & Magnuson, K. (2003). Off with Hollingshead: Socioeconomic resources, parenting, and child development. In M. H. Bornstein & R. H. Bradley (Eds.), Socioeconomic status, parenting, and child development (pp. 83–106). Lawrence Erlbaum Associates Publishers.

[bib22] Duncan, G. J. , & Magnuson, K. (2012). Socioeconomic status and cognitive functioning: Moving from correlation to causation. Wiley Interdisciplinary Reviews: Cognitive Science, 3(3), 377–386. 10.1002/wcs.1176, 26301469

[bib23] Dunn, L. M. , & Dunn, D. M. (2007). PPVT-4: Peabody picture vocabulary test (4th ed.). Pearson Assessments. 10.1037/t15144-000

[bib24] Elleman, A. M. , Oslund, E. L. , Griffin, N. M. , & Myers, K. E. (2019). A review of middle school vocabulary interventions: Five research-based recommendations for practice. Language, Speech, and Hearing Services in Schools, 50(4), 477–492. 10.1044/2019_LSHSS-VOIA-18-0145, 31600468

[bib25] Ensminger, M. E. , & Fothergill, K. E. (2014). A decade of measuring SES: What it tells us and where to go from here. In M. H. Bornstein & R. H. Bradley (Eds.), Socioeconomic Status, Parenting, and Child Development (pp. 13–27). New York: Psychology Press.

[bib26] Entwislea, D. R. , & Astone, N. M. (1994). Some practical guidelines for measuring youth’s race/ethnicity and socioeconomic status. Child Development, 65(6), 1521–1540. 10.1111/J.1467-8624.1994.TB00833.X

[bib27] Fedorenko, E. , Scott, T. L. , Brunner, P. , Coon, W. G. , Pritchett, B. , Schalk, G. , & Kanwisher, N. (2016). Neural correlate of the construction of sentence meaning. Proceedings of the National Academy of Sciences of the United States of America, 113(41), E6256–E6262. 10.1073/pnas.1612132113, 27671642 PMC5068329

[bib28] Fenson, L. , Dale, P. S. , Reznick, J. S. , Bates, E. , Thal, D. J. , Pethick, S. J. , Tomasello, M. , Mervis, C. B. , & Stiles, J. (1994). Variability in early communicative development. Monographs of the Society for Research in Child Development, 59(5). 10.2307/1166093 7845413

[bib29] Fernald, A. , Gruter, T. , Hurtado, N. , & Marchman, A. (2013). Relative language exposure, processing efficiency and vocabulary in Spanish–English bilingual toddlers. Bilingualism: Language and Cognition, 17(1), 189–202. 10.1017/S136672891300014X 35720734 PMC9206230

[bib30] Fukkink, R. G. , Blok, H. , & De Glopper, K. (2001). Deriving word meaning from written context: A multicomponential skill. Language Learning, 51(3), 477–496. 10.1111/0023-8333.00162

[bib31] Gersten, R. , Dimino, J. , Jayanthi, M. , Kim, J. S. , & Santoro, L. E. (2010). Teacher study group: Impact of the professional development model on reading instruction and student outcomes in first grade classrooms. American Educational Research Journal, 47(3), 694–739. 10.3102/0002831209361208

[bib32] Hagoort, P. , Hald, L. , Bastiaansen, M. , & Petersson, K. M. (2004). Integration of word meaning and world knowledge in language comprehension. Science, 304(5669), 438–441. 10.1126/science.1095455, 15031438

[bib33] Harding, J. F. , Morris, P. A. , & Hughes, D. (2015). The relationship between maternal education and children’s academic outcomes: A theoretical framework. Journal of Marriage and Family, 77(1), 60–76. 10.1111/jomf.12156

[bib34] Harmony, T. , Alvarez, A. , Pascual, R. , Ramos, A. , Marosi, E. , Díaz De León, A. E. , Valdés, P. , & Becker, J. (1988). EEG maturation on children with different economic and psychosocial characteristics. International Journal of Neuroscience, 41(1–2), 103–113. 10.3109/00207458808985747, 3410648

[bib35] Heckman, J. J. (2006). Skill formation and the economics of investing in disadvantaged children. Science, 312(5782), 1900–1902. 10.1126/science.1128898, 16809525

[bib37] Hoff, E. (2003). Causes and consequences of SES-related differences in parent-to-child speech. In M. H. Bornstein & R. H. Bradley (Eds.), Socioeconomic status, parenting, and child development (pp. 147–160). Lawrence Erlbaum Associates Publishers.

[bib36] Hoff, E. , Core, C. , Place, S. , Rumiche, R. , Señor, M. , & Parra, M. (2012). Dual language exposure and early bilingual development. Journal of Child Language, 39(1), 1. 10.1017/S0305000910000759, 21418730 PMC4323282

[bib38] Hoffman, L. (2003). Methodological issues in studies of SES, parenting, and child development. In M. H. Bornstein & R. H. Bradley (Eds.), Socioeconomic status, parenting, and child development (pp. 125–143). Lawrence Erlbaum Associates Publishers.

[bib39] Kielar, A. , Meltzer, J. A. , Moreno, S. , Alain, C. , & Bialystok, E. (2014). Oscillatory responses to semantic and syntactic violations. Journal of Cognitive Neuroscience, 26(12), 2840–2862. 10.1162/jocn_a_00670, 24893735

[bib40] Kielar, A. , Panamsky, L. , Links, K. A. , & Meltzer, J. A. (2015). Localization of electrophysiological responses to semantic and syntactic anomalies in language comprehension with MEG. NeuroImage, 105, 507–524. 10.1016/j.neuroimage.2014.11.016, 25463470

[bib41] Levine, D. , Pace, A. , Luo, R. , Hirsh-Pasek, K. , Golinkoff, R. M. , de Villiers, J. , Iglesias, A. , & Wilson, M. S. (2020). Evaluating socioeconomic gaps in preschoolers’ vocabulary, syntax, and language process skills with the Quick Interactive Language Screener (QUILS). Early Childhood Research Quarterly, 50, 114–128. 10.1016/j.ecresq.2018.11.006

[bib42] Lewis, A. G. , & Bastiaansen, M. (2015). A predictive coding framework for rapid neural dynamics during sentence-level language comprehension. Cortex, 68, 155–168. 10.1016/j.cortex.2015.02.014, 25840879

[bib43] Lewis, A. G. , Wang, L. , & Bastiaansen, M. (2015). Fast oscillatory dynamics during language comprehension: Unification versus maintenance and prediction?, Brain and Language, 148, 51–63. 10.1016/j.bandl.2015.01.003, 25666170

[bib44] Luo, R. , Pace, A. , Levine, D. , Iglesias, A. , de Villiers, J. , Golinkoff, R. M. , Wilson, M. S. , & Hirsh-Pasek, K. (2021). Home literacy environment and existing knowledge mediate the link between socioeconomic status and language learning skills in dual language learners. Early Childhood Research Quarterly, 55, 1–14. 10.1016/j.ecresq.2020.10.007

[bib45] Maguire, M. J. , & Schneider, J. M. (2019). Socioeconomic status related differences in resting state EEG activity correspond to differences in vocabulary and working memory in grade school. Brain and Cognition, 137, 103619. 10.1016/j.bandc.2019.103619, 31655309

[bib46] Maguire, M. J. , Schneider, J. M. , Middleton, A. E. , Ralph, Y. , Lopez, M. , Ackerman, R. A. , & Abel, A. D. (2018). Vocabulary knowledge mediates the link between socioeconomic status and word learning in grade school. Journal of Experimental Child Psychology, 166, 679–695. 10.1016/j.jecp.2017.10.003, 29103588

[bib47] Maris, E. , & Oostenveld, R. (2007). Nonparametric statistical testing of EEG- and MEG-data. Journal of Neuroscience Methods, 164(1), 177–190. 10.1016/j.jneumeth.2007.03.024, 17517438

[bib48] Mestres-Missé, A. , Rodriguez-Fornells, A. , & Münte, T. F. (2007). Watching the brain during meaning acquisition. Cerebral Cortex, 17(8), 1858–1866. 10.1093/cercor/bhl094, 17056648

[bib49] Nagy, W. E. , Herman, P. A. , & Anderson, R. C. (1985). Learning words from context. Reading Research Quarterly, 20(2), 233. 10.2307/747758

[bib50] Nagy, W. , & Townsend, D. (2012). Words as tools: Learning academic vocabulary as language acquisition. Reading Research Quarterly, 47(1), 91–108. 10.1002/RRQ.011

[bib51] Oldfield, R. C. (1971). The assessment and analysis of handedness: the Edinburgh inventory. Neuropsychologia, 9(1), 97–113. 10.1016/0028-3932(71)90067-4, 5146491

[bib52] Oostenveld, R. , Fries, P. , Maris, E. , & Schoffelen, J. M. (2011). FieldTrip: Open source software for advanced analysis of MEG, EEG, and invasive electrophysiological data. Computational Intelligence and Neuroscience, 2011, 156869. 10.1155/2011/156869, 21253357 PMC3021840

[bib53] Otero, G. A. , Pliego-Rivero, F. B. , Fernández, T. , & Ricardo, J. (2003). EEG development in children with sociocultural disadvantages: A follow-up study. Clinical Neurophysiology, 114(10), 1918–1925. 10.1016/S1388-2457(03)00173-1, 14499754

[bib54] Pace, A. , Alper, R. , Burchinal, M. R. , Golinkoff, R. M. , & Hirsh-Pasek, K. (2019). Measuring success: Within and cross-domain predictors of academic and social trajectories in elementary school. Early Childhood Research Quarterly, 46, 112–125. 10.1016/j.ecresq.2018.04.001

[bib55] Pace, A. , Luo, R. , Levine, D. , Iglesias, A. , de Villiers, J. , Golinkoff, R. M. , Wilson, M. S. , & Hirsh-Pasek, K. (2021). Within and across language predictors of word learning processes in dual language learners. Child Development, 92(1), 35–53. 10.1111/cdev.13418, 32776574

[bib56] Prystauka, Y. , & Lewis, A. G. (2019). The power of neural oscillations to inform sentence comprehension: A linguistic perspective. Language and Linguistics Compass, 13(9), e12347. 10.1111/lnc3.12347, 33042211 PMC7546279

[bib57] Ralph, Y. K. , Schneider, J. M. , Abel, A. D. , & Maguire, M. J. (2020). Using the N400 event-related potential to study word learning from context in children from low- and higher-socioeconomic status homes. Journal of Experimental Child Psychology, 191, 104758. 10.1016/j.jecp.2019.104758, 31855830 PMC8191850

[bib58] Rice, M. (1990). Preschooler’s QUIL: Quick incidental learning of words. In G. Conti-Ramsden & C. E. Snow (Eds.), Children’s Language (7th ed., pp. 171–195). Erlbaum.

[bib59] Rice, M. L. , Buhr, J. , & Oetting, J. B. (1992). Specific-language-impaired children’s quick incidental learning of words. Journal of Speech, Language, and Hearing Research, 35(5), 1040–1048. 10.1044/jshr.3505.1040, 1447916

[bib60] Richels, C. G. , Johnson, K. N. , Walden, T. A. , & Conture, E. G. (2013). Socioeconomic status, parental education, vocabulary and language skills of children who stutter. Journal of Communication Disorders, 46(4), 361–374. 10.1016/j.jcomdis.2013.07.002, 23906898 PMC3880199

[bib61] Sauseng, P. , Klimesch, W. , Doppelmayr, M. , Pecherstorfer, T. , Freunberger, R. , & Hanslmayr, S. (2005). EEG alpha synchronization and functional coupling during top-down processing in a working memory task. Human Brain Mapping, 26(2), 148–155. 10.1002/hbm.20150, 15929084 PMC6871735

[bib62] Schneider, J. M. , Abel, A. D. , Ogiela, D. A. , McCord, C. , & Maguire, M. J. (2018). Developmental differences in the neural oscillations underlying auditory sentence processing in children and adults. Brain and Language, 186, 17–25. 10.1016/j.bandl.2018.09.002, 30199760

[bib65] Schneider, J. M. , Abel, A. D. , Ogiela, D. A. , Middleton, A. E. , & Maguire, M. J. (2016). Developmental differences in beta and theta power during sentence processing. Developmental Cognitive Neuroscience, 19, 19–30. 10.1016/j.dcn.2016.01.001, 26774879 PMC6988103

[bib63] Schneider, J. M. , & Maguire, M. J. (2018a). Developmental differences in the neural correlates supporting semantics and syntax during sentence processing. Developmental Science, 22(4), e12782. 10.1111/desc.12782, 30525288

[bib64] Schneider, J. M. , & Maguire, M. J. (2018b). Identifying the relationship between oscillatory dynamics and event-related responses. International Journal of Psychophysiology, 133, 182–192. 10.1016/J.IJPSYCHO.2018.07.002, 29981766

[bib66] Schwab, J. F. , & Lew-Williams, C. (2016). Language learning, socioeconomic status, and child-directed speech. Wiley Interdisciplinary Reviews: Cognitive Science, 7(4), 264–275. 10.1002/wcs.1393, 27196418 PMC5901657

[bib67] Shavlik, M. , Davis-Kean, P. E. , Schwab, J. F. , & Booth, A. E. (2020). Early word-learning skills: A missing link in understanding the vocabulary gap? Developmental Science, 24(2), e13034. 10.1111/desc.13034, 32881178

[bib68] Spencer, E. J. , & Schuele, C. M. (2012). An examination of fast mapping skills in preschool children from families with low socioeconomic status. Clinical Linguistics & Phonetics, 26(10), 845–862. 10.3109/02699206.2012.705215, 22954365

[bib69] Storkel, H. L. (2013). A corpus of consonant–vowel–consonant real words and nonwords: Comparison of phonotactic probability, neighborhood density, and consonant age of acquisition. Behavior Research Methods, 45(4), 1159–1167. 10.3758/s13428-012-0309-7, 23307574 PMC3633677

[bib70] Texas Demographic Center. (n.d.). Retrieved February 11, 2021, from https://demographics.texas.gov/

[bib71] Tomalski, P. , Moore, D. G. , Ribeiro, H. , Axelsson, E. L. , Murphy, E. , Karmiloff-Smith, A. , Johnson, M. H. , & Kushnerenko, E. (2013). Socioeconomic status and functional brain development–associations in early infancy. Developmental Science, 16(5), 676–687. 10.1111/desc.12079, 24033573

[bib72] Uhlhaas, P. J. , Roux, F. , Rodriguez, E. , Rotarska-Jagiela, A. , & Singer, W. (2010). Neural synchrony and the development of cortical networks. Trends in Cognitive Sciences, 14(2), 72–80. 10.1016/j.tics.2009.12.002, 20080054

[bib73] U.S. Census Bureau. (n.d.). Poverty thresholds. Retrieved February 11, 2021, from https://www.census.gov/data/tables/time-series/demo/income-poverty/historical-poverty-thresholds.html

[bib74] Vanderwert, R. E. , Zeanah, C. H. , Fox, N. A. , & Nelson III, C. A. (2016). Normalization of EEG activity among previously institutionalized children placed into foster care: A 12-year follow-up of the Bucharest Early Intervention Project. Developmental Cognitive Neuroscience, 17, 68–75. 10.1016/j.dcn.2015.12.004, 26724564 PMC4727988

[bib75] Wagovich, S. A. , Pak, Y. , & Miller, M. D. (2012). Orthographic word knowledge growth in school-age children. American Journal of Speech-Language Pathology, 21(2), 140–153. 10.1044/1058-0360(2012/10-0032), 22411772

[bib76] Wang, L. , Jensen, O. , van den Brink, D. , Weder, N. , Schoffelen, J.-M. , Magyari, L. , Hagoort, P. , & Bastiaansen, M. (2012). Beta oscillations relate to the N400m during language comprehension. Human Brain Mapping, 33(12), 2898–2912. 10.1002/hbm.21410, 22488914 PMC6870343

[bib77] Wang, L. , Zhu, Z. , & Bastiaansen, M. (2012). Integration or predictability? A further specification of the functional role of gamma oscillations in language comprehension. Frontiers in Psychology, 3, 187. 10.3389/fpsyg.2012.00187, 22701443 PMC3372880

[bib78] Weisleder, A. , & Fernald, A. (2013). Talking to children matters: Early language experience strengthens processing and builds vocabulary. Psychological Science, 24(11), 2143–2152. 10.1177/0956797613488145, 24022649 PMC5510534

[bib79] Wiederholt, J. L. , & Bryant, B. R. (2012). Gray oral reading test: Examiner’s record booklet; form-A (5th ed.). Pro-Ed.

[bib80] Winkler, I. , Brandl, S. , Horn, F. , Waldburger, E. , Allefeld, C. , & Tangermann, M. (2014). Robust artifactual independent component classification for BCI practitioners. Journal of Neural Engineering, 11(3), 35013. 10.1088/1741-2560/11/3/035013, 24836294

[bib81] Winkler, I. , Haufe, S. , & Tangermann, M. (2011). Automatic classification of artifactual ICA-components for artifact removal in EEG signals. Behavioral and Brain Functions, 7(1), 1. 10.1186/1744-9081-7-30, 21810266 PMC3175453

