# Supplementary material for: Neural Oscillations Reveal Differences in the Process of Word Learning among School-Aged Children from Lower Socioeconomic Status Backgrounds
Source: Neurobiol Lang (Camb). 2021 Jul 13;2(3):372–88. doi: 10.1162/nol_a_00040 (PMC8386290; doi:10.1162/nol_a_00040)
Supplement: Supplementary file 1 [file nol-2-3-372-s001.docx]

*Supplementary Table 1.* Additional demographic information for children classified as better or worse learners. Reading and math readiness, as well as percent of children who are economically disadvantaged, were determined by school averages across grades, rather than individual scores.

|  | **Better Learners** | **Worse Learners** | **p-value** |
| --- | --- | --- | --- |
| *N* | 22 | 22 |  |
| **School Environment** |  |  |  |
| Reading Readiness  [mean (SD)] | 39.10 (14.53) | 42.75 (22.60) | 0.65 |
| Math Readiness  [mean (SD)] | 42.80 (17.26) | 42.25 (21.77) | 0.95 |
| Percent of Children who are Economically Disadvantaged  [mean (SD)] | 61.05% (32.56%) | 78.83% (27.65%) | 0.11 |
| **Living Conditions** |  |  |  |
| Number of families who rent | 14 | 16 | 0.66 |
| Total members in household  [mean (SD)] | 3.09 (1.15) | 3.59 (0.91) | 0.12 |
| Birth Order  [mean (SD)] | 1.82 (0.96) | 1.68 (0.72) | 0.596 |
| Number of Books in the Home |  |  | 0.79 |
| 0-10 | 1 | 1 |  |
| 11-100 | 17 | 16 |  |
| 101-500 | 1 | 4 |  |
| 500+ | 1 | 0 |  |
| NA | 2 | 1 |  |
| Hours slept the night before testing [mean (SD)] | 8.30 (1.21) | 9.02 (1.20) | 0.06 |

*Supplementary Figure 1.* Mean and median of dB power between better and worse learners. While better learners demonstrate a stronger correlation between central measures of power (*R* = .71), a moderate to strong relationship was still present in worse learners (*R* = .51).

*Supplementary Figure 2.* The reliability of frequency-band-specific power averaged over all trials.
